# An informed shared decision making programme on the prevention of myocardial infarction for patients with type 2 diabetes in primary care: protocol of a cluster randomised, controlled trial

**DOI:** 10.1186/s12875-015-0257-2

**Published:** 2015-03-31

**Authors:** Susanne Buhse, Ingrid Mühlhauser, Nadine Kuniss, Ulrich Alfons Müller, Thomas Lehmann, Katrin Liethmann, Matthias Lenz

**Affiliations:** Faculty of Mathematics, Informatics and Natural Sciences, Unit of Health Sciences and Education, University of Hamburg, Martin-Luther-King-Platz 6, Hamburg, 20146 Germany; Department of Internal Medicine III, Endocrinology and Metabolic Diseases, University Hospital Jena, Jena, Germany; Centre for Clinical Studies, University Hospital Jena, Jena, Germany

**Keywords:** Diabetes mellitus, Type 2, Myocardial infarction, Primary prevention, Patient education, Patient participation, Shared decision making, Evidence-based medicine, Primary care

## Abstract

**Background:**

International and national societies claim a patient centred approach including shared decision making (SDM) in diabetes care. In a previous project, a SDM programme on the prevention of myocardial infarction has been developed. It is aimed at supporting patients with type 2 diabetes to make informed choices on preventive options, to share the decision making process with the health care team, and to improve adherence to the chosen treatment. In this study, the programme will be implemented and evaluated in primary care practices.

**Methods/Design:**

A cluster randomised, controlled trial will be conducted to compare the SDM programme with standard care enrolling patients with type 2 diabetes (N = 306) from primary care practices (N = 24). The intervention programme comprises a six hours provider training, a patient decision aid including evidence-based information, a 90 minutes structured teaching session provided by medical assistants, a sheet to document the patients’ individual treatment goals, and a structured consultation with the general practitioner for sharing information, setting treatment goals, and for adapting treatment regimens if necessary. Patients in the control group receive a brief extract of recommendations of the German National Disease Management Guideline on the treatment of patients with type 2 diabetes. Primary outcome measure is adherence to blood pressure treatment and statin treatment at 6 months follow-up. Secondary outcome measures comprise informed choice and the achievement of patients’ treatment goals. Analyses will be carried out on intention-to-treat basis. Concurrent qualitative methods will be used to explore the implementation processes.

**Discussion:**

At the end of this study, information on the efficacy of the SDM programme in the primary care context will be available. In addition, processes that might interfere with or that might promote a successful implementation will be identified.

**Trial registration:**

ISRCTN77300204.

## Background

Premature cardiovascular morbidity and mortality is the main health risk for people with type 2 diabetes. An array of preventive interventions is recommended to the patients, such as increasing exercise, changing eating habits, quitting smoking, and taking various drugs [1]. Evidence on the efficacy of the recommended measures is variable. Some may even do more harm than good [2]. Patients frequently feel demotivated and overloaded by the plethora of medical prescriptions. This might contribute to poor long-term adherence [3,4] even to the most effective preventive interventions such as blood pressure control [5] and use of statins [6]. Lack of patient involvement in decision making has been suggested as an important reason for weak adherence and limited treatment success [7].

Since 2012 the American Diabetes Association (ADA) and the European Association for the Study of Diabetes (EASD) claim a “patient centred approach” in the care of people with type 2 diabetes [8], including self-management and shared decision making (SDM). In SDM patients and health care providers simultaneously participate in the decision making process, by sharing information and jointly negotiating a treatment to implement [9]. In Germany, the implementation of SDM in the clinical encounter is explicitly recommended by the German Medical Association and Scientific Medical Societies [1].

There is reasonable evidence supporting the effectiveness of SDM. Involvement of patients can improve knowledge [10-12]. And, better-informed patients involve themselves more in their own medical decisions [13-15].

Though explicitly recommended, SDM has not yet been implemented in diabetes care [16,17]. There are several barriers of implementation encompassing *organisational factors* such as time, setting, and workflow as well as *decision making interaction factors* such as patient characteristics and power imbalance in the patient-physician relationship [18,19]. Sustainable care in chronic conditions such as diabetes requires the involvement of family members and the healthcare team. Légaré et al. [20] proposed an interprofessional SDM model, in which the individual patient follows a coached process to make informed, value-based decisions in concert with an interprofessional healthcare team.

Successful SDM can only be achieved if patients understand disease risks and probabilities of benefits and harms of healthcare options and interpret them in a realistic way [10,12,21,22]. However, patients and care providers often have difficulties to comprehend this sort of information [23,24]. Thus, evidence-based patient information (EBPI) is needed comprising relevant and reliable information explained and designed in an understandable manner. EBPI is typically provided in form of a patient decision aid (DA) [25].

Internationally, there are different concepts aimed at facilitating SDM [26]. Some of them are generic others disease specific. Implementation in health care settings may require translation and adaptation to specific conditions. The “Inventory of Shared Decision Making Programs for Healthcare Professionals” [26] lists five German SDM-programmes. Four of them are published [27-30]. Three of them address SDM in a rather generic way. They do not include specific EBPI [27,29,30]. DoktormitSDM [30] however, includes a training for physicians using exemplary EBPI on immunotherapy in multiple sclerosis. The ARRIBA programme [28] targets cardiovascular prevention but is not specifically designed for patients with diabetes. There is a diabetes specific extension available [31], which focuses on oral antidiabetic drugs and conventional and intensified insulin therapy. Only one programme [29] includes an interprofessional approach, by explicitly addressing the cooperation and communication within the team.

In our recent project, an informed shared decision making (ISDM) programme on the prevention of myocardial infarction in type 2 diabetes has been developed [32,33]. The concept includes an evidence-based DA and a corresponding teaching module provided by diabetes educators.

The ISDM programme has been evaluated in a randomised, controlled trial (RCT) [34] under high fidelity conditions in an outpatient setting at the Department for Endocrinology and Metabolic Diseases of the University Hospital Jena, Germany. Follow-up data is currently being collected. Primary endpoint has been patients’ knowledge regarding the individual heart attack risk, benefits and harms of preventive options. Secondary endpoints have been realistic expectations and achievement of treatment goals. Preliminary data assessed after the teaching sessions are available [35]. Patients allocated to the ISDM programme achieved higher scores of knowledge and higher scores of realistic expectations. ISDM patients more often prioritised an achievement of their blood pressure goals. Blood glucose goals were prioritised by 32.9% of the ISDM group and by 60% of the control group. Prescriptions of statins did not change from baseline to post teaching, but individual choice to not taking statins indicated changes of adherence to statin medication.

In sum, the study showed that the ISDM programme has impact on patients’ knowledge and prioritisation. However, it may not realign power-imbalance between patients and their health care team. Hence, the ISDM programme has been augmented by including an additional training module for physicians to facilitate implementation of SDM.

The aim of the planned cluster randomised, controlled trial is to evaluate the efficacy of the ISDM programme in the primary care context. Alongside to this trial, the implementation process will be evaluated using qualitative research methods.

The reporting of this study follows current statements [36,37].

## Objectives

The key hypothesis is that patients who actively participate in decision making show better adherence to their individual treatment goals. We primarily investigate if patients allocated to the ISDM group are more likely to adhere to their antihypertensive or statin medication. We also investigate if patients in the ISDM group achieve their HbA1c- and blood pressure goals with fewer drug prescriptions and if they achieve lower levels of HbA1c, blood pressure, or cholesterol with unchanged or fewer prescriptions.

We also hypothesise that patients in the ISDM group less frequently prioritise intensified glucose control and more frequently blood pressure control and statin treatment.

Further objectives are to assess if the ISDM group achieves a better understanding and higher levels of realistic expectations regarding heart attack risk and probabilities of benefits and harms of preventive options.

Alongside to the trial, processes and barriers of implementation will be qualitatively assessed. We expect that the implementation of the ISDM programme in primary care will be perceived as feasible. Patients will be perceived as more competent. The workload will not be perceived as more stressful and time consuming.

## Methods

### Design

A parallel group, patient- and assessor-blinded, cluster randomised, controlled trial with six months follow-up will be conducted. Alongside the trial, qualitative methods will be used to achieve in-depth understanding of implementation processes.

### Setting

The study will take place in Germany, predominantly in the Free State of Thuringia and partly in Saxony-Anhalt and Hamburg. Study setting is primary care practices (cluster), some are specialised on patients with diabetes. Practices are eligible that participate in the German Disease Management Programme (DMP) for type 2 diabetes.

DMPs have been introduced in Germany to improve care of patients with chronic diseases [38]. Main objective is a holistic and structured care mainly provided by family practices. DMPs are based on evidence-based guidelines and are approved by the German Federal Insurance Authority. Between 83% and 94% of patients with type 2 diabetes are being inscribed in the DMP [39]. Patients are followed quarterly. At these visits parameters of care have to be systematically documented on treatment goals, participation in patient education programmes, prescribed medication, some laboratory results and hypoglycaemic events. The quality of the DMP is evaluated by the Central Research Institute of Ambulatory Health Care (ZI) and institutes commissioned by health insurances. The ZI [40] also organises the education of trainers qualified to train general practitioners (GPs) and medical assistants to deliver structured treatment and teaching programmes. Various treatment and teaching programmes are approved, e.g. for patients with non-insulin-dependent or with insulin-dependent diabetes mellitus and patients with intensive insulin treatment [41-44]. The programmes aim at fostering self-management. They comprise teaching modules for small groups of patients and typically encompass 4 to 12 subsequent sessions.

### Eligibility and recruitment

Primary care practices are eligible, if they employ at least one diabetes educator or a medical assistant (MA) or a nurse with further training in structured diabetes education and if they provide structured teaching and treatment within the DMP for patients with type 2 diabetes. Practices will be invited for participation by sending a brief letter of information and personal contact. Before randomisation, a project member will visit each participating practice to provide detailed information on the study and to support patient recruitment.

**Figure 1:**
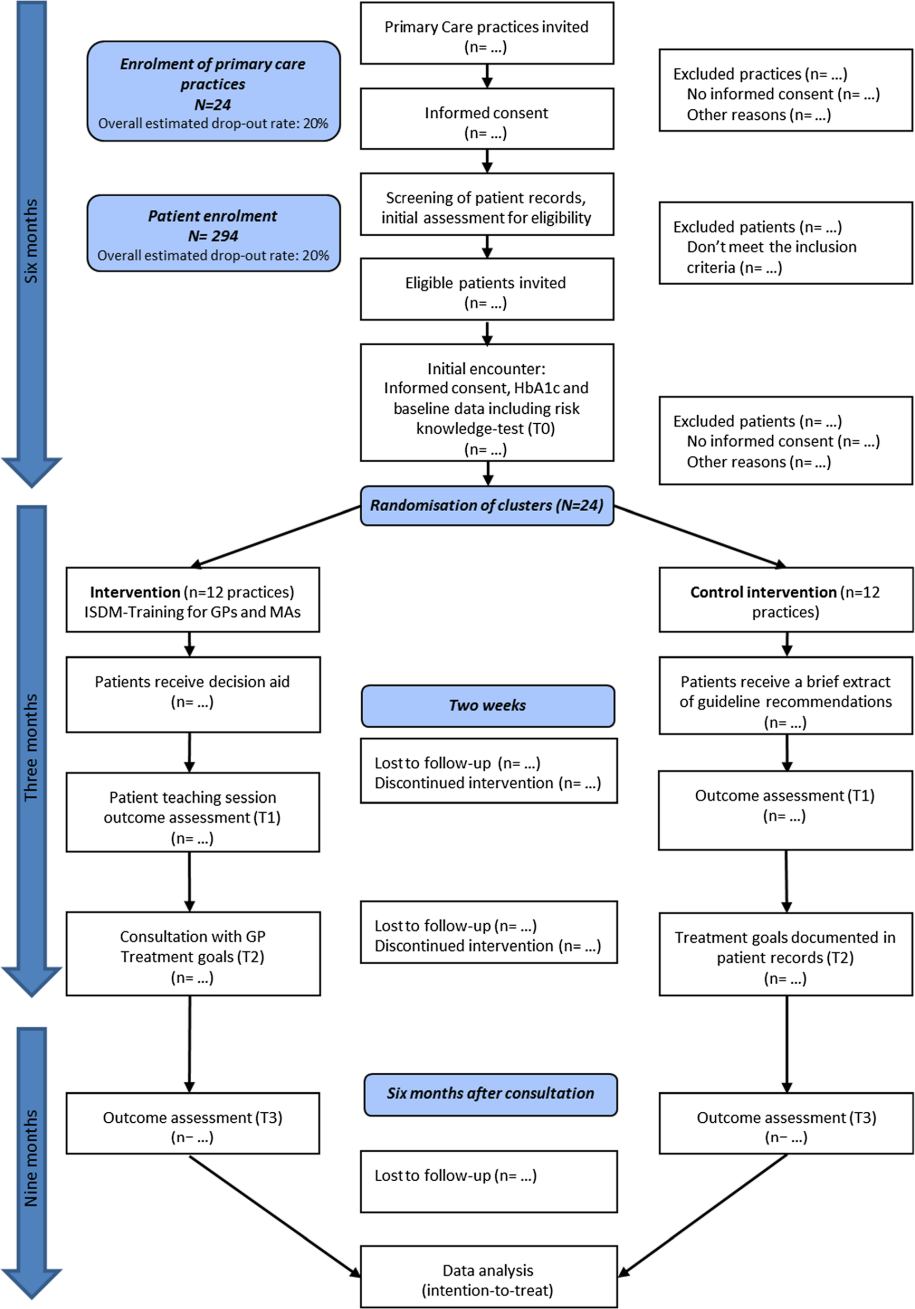
**Study flow.** ISDM = Informed Shared Decision Making; GP = General Practitioner; MA = Medical Assistant; T0 = data collection at baseline; T1 = data collection at the end of the teaching session; T2 = data collection during consultation; T3 = data collection at 6 months follow-up.

Patients are eligible if they meet the following criteria: age between 40 and 69 years, type 2 diabetes, HbA1c-value less than 9%, and previous participation in structured treatment and teaching sessions as they are typically provided within the DMP. Patients are excluded if they have a previous diagnosis of ischaemic heart disease (ICD I20-I25), stroke (ICD I63), proliferative retinopathy, chronic kidney disease stage 3 or higher [45], metastatic cancer, are addicted to alcohol, or cared by a legal guardian. Supported by a research associate, MA or GP of each practice will screen the patient records for eligibility (study flow Figure 1). Eligible patients will be informed and asked for participation.

### Randomisation and blinding

Randomisation of practices will be performed after patient recruitment and retrieval of baseline data. To ensure close balance of entities in each group, randomisation will be performed in blocks of 4. The randomisation sequence will be generated by the Centre for Clinical Studies at the University Hospital Jena. Blinding of practices that deliver the intervention programme is not feasible. However, they will be asked to keep the allocation blinded as far as possible, i.e. to not reveal study details to the patients. Patients will be informed that the objective of the study is to compare two different approaches to better acknowledge patients’ preferences and values in decision making on preventive measures and that they have an equal chance of receiving the intervention or the control programme. Allocation concealment will be ensured for database entry and statistical analyses.

### Study interventions

The ISDM programme is a complex intervention [46-49]. By definition, complex interventions comprise interdependently acting components essential to their proper functioning. E.g. the DA, the teaching module, and the provider training act interdependently. Each component was piloted within the target group and iteratively optimised [32,33]. An additional aspect of complexity is the implementation context, characterised by the setting, the providers, whether those are able and willing to implement the SDM concept, and all factors influencing their ability and willingness. In order to simplify implementation, duration and structure have been adapted to current teaching modules [41,42]. The underlying approach follows Ajzen’s theory of planned behaviour [50], which suggests behaviour is influenced by 1) individual attitudes, 2) subjective norms such as perceived attitudes of family members or individuals of the health care team, and 3) perceived and actual individual behaviour control. The most predictive variable of SDM behaviour is intention [51]. Subjective norms and perceived behaviour control are the most frequently identified determinants of a health professional’s intention to perform SDM. Subjective norms will be addressed by involving the whole team of each practice. Providing evidence-based information aims at strengthening behaviour control by resolving knowledge deficits and by realigning unrealistic expectations.

#### Intervention group (ISDM programme)

The intervention programme (Table 1) comprises (1) an evidence-based patient DA [33] on the prevention of myocardial infarction, (2) a structured teaching module [32] provided by MAs, (3) a sheet to document the individual treatment goals, (4) a six hours provider training, and (5) an organisational study meeting with the personnel in the participating practice.

The DA includes evidence-based patient information on heart attack risk, risk factors, and different preventive options in an understandable manner [33]. In the development process, systematic literature search was performed for all recommended preventive options [33]. Information on the efficacy of preventive treatment has only been extracted from rigid RCT or high quality systematic reviews.

The DA guides users to estimate their individual risk of having a myocardial infarction in the next ten years and how it can be reduced by statin treatment. It provides information about the probable effects of more or less intensified glucose control and blood pressure control on the combined diabetes-related endpoint as used in the UKPDS [5,52].

The DA visualises text-based risk information by using combinations of 100-stick figure pictograms and bar graphs. An appendix provides additional information, e.g. reliability of individual risk prognosis. Important technical and medical terms are explained in a glossary.

The patient teaching module is curriculum-led and focuses on the EBPI provided in the DA. A teaching session targets groups of 4 to 6 patients and takes about 90 minutes. Two weeks before a session, the patients are asked to read and work through the DA [33]. During a session, the MA guides the patients through the decision making process. This encompasses assessing each patient’s individual heart attack risk, providing outcome probabilities of the available preventive treatment options, and supporting patients to set individual goals regarding smoking cessation, glucose control, blood pressure control, and statin treatment. Each patient notes individual preferences on treatment goals and the prioritised goal on a documentation sheet. Based on this, the patient and the GP deliberate and agree on treatment goals (Figure 1). This agreement is fixed on the documentation sheet for the patient to be taken home and documented in the patient record. The sheet can also supplement the patients’ diabetes management diary.

A weakness of the ISDM programme in the pilot RCT [35] was that the health care teams were not trained in SDM. They may not have explicitly invited patients to share their attitudes and preferences and to actively participate in decision making. Power imbalance [19] might still have been a barrier. To address this issue, we developed two additional components: The sheet for the documentation of the treatment goals and a SDM training for the GPs. Both are aimed at optimising the consultation in terms of SDM. The documentation of treatment goals on the sheet is intended to focus the consultation.

During this study, research fellows and a psychologist give the SDM training. Initially, the health care teams receive detailed information about contents and structure of the ISDM programme and SDM basics. The GPs are trained in re-focusing the consultation in terms of SDM. The MAs are trained to provide the patient teaching in a standardised way by following the curriculum. Contents of the curriculum are delivered in didactic lectures, teaching situations are simulated in role plays. Finally, to ensure and to reflect training success, the MAs work through a knowledge test and discuss the results.

#### Control group

In the control group, standard care is provided. It is defined as usual care augmented by a brief extract of the patients’ version of the German National Disease Management Guideline on the treatment of patients with type 2 diabetes [53] with a link to the full version of the guideline. In addition, organisational study meetings with the health care teams of the participating practices are conducted (Table 1). In Germany, scientific medical societies in cooperation with patient representatives develop so called *patient guidelines* of each National Disease Management Guideline. Patient guidelines comprise the main aspects of the medical guideline in a concise and comprehensible manner. The patient guideline on the treatment of type 2 diabetes includes information on the diagnosis and therapy of type 2 diabetes as well as recommendations regarding blood glucose and blood pressure targets, cholesterol values, and lifestyle interventions. The control intervention does not include an additional teaching module, but the patients have the opportunity to ask their health care professionals for more information. Patients are asked to set individual treatment goals. After completion of the study the ISDM programme will also be offered to the control group.

**Table 1:** **Characteristics of the ISDM intervention**

**Components**	**ISDM programme**	**Control intervention**
**Organisational meetings in the practices**	Participants: GPs and the MA of the participating practices	Participants: GPs and the MA of the participating practices
	Duration: one hour	Duration: one hour
	Core element: Study organisational meeting	Core element: Study organisational meeting
**Training for the providers**	Participants: n = 4 to 6 GPs, plus the MA employed in the participating practices	n.a.
	Duration: six hours	
	Core elements: the concept of SDM, curriculum for patient teaching, didactic lectures and role playing	
**Information for patients**	Topic: DA on the prevention of myocardial infarction in type 2 diabetes [33].	Topic: Brief extract of the German National Disease Management Guideline on the treatment of patients with type 2 diabetes, patients’ version [53]
	Date of delivery: 2 weeks before teaching session	Date of delivery: 2 weeks before practice visit
	Core elements: Evidence-based patient information on heart attack risk, risk factors, and different preventive options, combinations of 100-stick figure pictograms and bar graphs, and user guide for risk estimation	Core elements: Recommendations related to treatment targets and a link to the full version of the guideline [53]
**Patient teaching module**	Participants: n = 4 to 6 patients per group	n.a.
	Duration: 90 minutes	
	Core elements: DA on the prevention of myocardial infarction [33], corresponding curriculum and media	
**Consultation with GP**	Duration: Approx. 10 minutes	n.a.
	Core element: Sheet for the documentation of individual treatment goals	

GPs = General Practitioners, MA = Medical Assistant, SDM = Shared Decision Making, DA = Decision Aid, n.a. = not applicable.

### Outcomes

According to our key study hypothesis, the most relevant outcome parameter would actually be the achievement of any preventive treatment goal prioritised by the individual patient. However, operationalization is not feasible in the context of this study. The parameter would encompass a variety of individually tailored goals and regimens regarding glucose control, blood pressure control, smoking secession, and cholesterol-lowering medication. In addition, HbA1c levels of the patients in both study arms of the pilot RCT were mostly already prioritised at baseline and adjusted at low levels. Blood pressure therapy was prescribed for patients with hypertension.

The ISDM programme is designed to initiate SDM. SDM is a multi-dimensional construct, which is challenging to evaluate. There is no gold standard to quantify the degree of patient involvement in SDM [54]. Available assessment instruments typically focus on the patient’s perspective [55]. An instrument that considers multiple perspectives [56] such as the patients’, the physicians’, or the observers’ perspective showed substantial inconsistency between them [57].

Sufficient knowledge is a prerequisite for adequately setting treatment goals in terms of SDM. The success of EBPI and SDM interventions is frequently evaluated by using the multi-dimensional parameter of *informed choice* [58]. This is characterised by adequate knowledge and consistency between attitude and uptake. However, *informed choice* cannot be used as a primary endpoint in our study since it is very unlikely that patients in the control group will make informed choices at all. Patients in the ISDM group of our pilot RCT [35] achieved adequate levels of knowledge, whereas patients without the ISDM programme did not.

Within the ISDM programme, patients are encouraged to make treatment choices consistent with individual attitudes and daily living. Thus, the chosen therapy may already be more adjusted to individual preferences. We hypothesise that patients are more adherent to medication, which is prescribed based on SDM. Hence, the primary outcome measure (Table 2) is defined as the adherence to blood pressure drug treatment or if not applicable the adherence to statin treatment. This primary outcome measure is operationalised as adherence to drug prescriptions at six months follow-up.

Secondary outcome measures are: 1) informed choice regarding statin treatment, blood pressure control, glucose control, and smoking cessation; 2) the achievement of treatment goals regarding statin treatment, blood pressure, HbA1c, and smoking cessation; 3) prioritisation of treatment goals; 4) realistic expectations [59] on the individual heart attack risk and on probabilities of benefits and harms of the available treatment options; 5) the level of patient knowledge and understanding relating to the concept of risk, the notion of heart attack risk, and the benefits and harms of preventive treatment.

Informed choice will be assessed equivalently to the *informed choice* construct developed by Marteau et al. [58]. To determine whether a patient makes an informed (or uninformed) choice, we assess and then combine three constructs: 1) knowledge relating to the concept of risk, the notion of heart attack risk, and the benefits and harms of preventive treatment; 2) the patient’s treatment goal; and 3) adherence to the decision.

Since an operationalization of prioritised goals is not feasible in the context of this study, we assess them separately. A categorisation of blood pressure and HbA1c goals in achieved vs. not achieved is not feasible at the individual level. Defining particular cut-off levels would be arbitrary. The achievement of these goals will be assessed by comparing group differences. Prioritised treatment goals will be described for each study group.

Additional outcome measures are change in clinical parameters (HbA1c, blood pressure, cholesterol) from baseline to follow-up and medication prescriptions. A previous RCT showed that patients who had participated in a structured hypertension self-management programme achieved lower blood pressure levels. At the same time prescription of antihypertensive medication decreased [60], which indicates better adherence to the prescribed medication. In the current cRCT, medication prescription will be interpreted in the light of the achievement of the corresponding treatment goals.

### Data collection

Patient records and standardised forms will be used to collect baseline characteristics and outcome data. Outcome data is documented electronically as scheduled in the DMP.

Baseline characteristics include age, gender, first-language (usually spoken at home), educational status, body mass index (BMI), smoking status, blood pressure, LDL-cholesterol level, total cholesterol level, HbA1c level, current medication, and previous participation in diabetes and hypertension patient education programmes. Individual baseline risks of myocardial infarction are calculated by using a risk assessment tool. The tool uses age, smoking status, and clinical parameters and is derived from the Framingham function calibrated [61] to the average heart attack risk in the German population.

The treatment goals set by the individual patient in the ISDM group will be assessed after the teaching session and after the consultation by extracting them from the documentation sheet. Treatment goals of the control group will be assessed at a practice visit by using a standardised documentation form.

Telephone interviews will be conducted to assess the adherence to the current drug prescriptions. A standardised interview guide will be developed including questions such as *“Patients with diabetes often need to take several pills. I am interested in your current use of pills. Could you tell me, which medication boxes do you use? Which substances are labelled on the boxes?”*, and “*Have you ever missed any pills in the past seven days?”* [62]*.* Patients are classified as non-adherent if their answers are inconsistent with the prescriptions. Patients without antihypertensive prescription will be asked about statin medication. Patients with neither antihypertensive nor statin prescription will not be included in data analysis for the primary endpoint. In order to avoid socially desirable answers, the interviews will be conducted by a study assistant of the study organising centre, independent from the practices and blinded towards the allocation. Interviews will be audio-taped. In ambiguous cases, the tape will be analysed by a project member who is also blinded toward patients’ allocation. Inter-rater consensus will be achieved by discussion. Cholesterol levels at baseline and follow-up will be used to validate data on adherence to statin choice.

Informed choice [58] will be assessed, by grouping the patients into one of eight classifications according to their knowledge (adequate if score above the median), treatment goal (e.g. taking statin or not), and adherence (to statin choice). Participants will be assigned as having made an informed choice e.g. on taking statins if they have adequate knowledge, set the goal to take a statin and are adherent to this decision at six months follow-up. An informed choice against e.g. taking statins occurs if a patient has adequate knowledge, set the goal to not take a statin and is adherent to this decision at six months follow-up. We consider patients who had inadequate knowledge or whose treatment goal is not consistent with patient’s behaviour at six months follow-up, to have made an uninformed choice.

Achievement of treatment goals regarding blood pressure and HbA1c levels will be assessed during the doctor’s appointments at follow-up. Smoking status will be assessed during the telephone interview at follow-up by using a standardised interview question: “*On how many of the past 30 days did you smoke a cigarette?*” If patients have smoked any cigarettes in the past month, they will be classified as smokers. Current blood pressure values and HbA1c levels will be extracted from patient records.

Knowledge and understanding will be assessed directly after the teaching session. A standardised questionnaire consisting of 11 questions will be used to assess both comprehension of risk information and realistic expectations. Item analysis was performed using data from 143 patients, who participated in the ongoing pilot RCT [35]. Values of item difficulty were on average 37 (Range: 6 – 58), indicating moderate to difficult item difficulties. Corrected item-total correlations regarding total knowledge test were on average 0.57 (Range: 0.35 – 0.73), which were acceptable values, too. Initially, the test consisted of 12 items, but one item was deleted due to weak item total correlation (0.29). Internal consistency was calculated for the total knowledge test (Cronbach’s alpha = 0.87) and for the subdomain of realistic expectations (Cronbach’s alpha = 0.86). In sum, these results indicate good item parameters.

Prescribed medication will be extracted from patient records.

At follow-up, patients will be asked to indicate which information format they believe they have received to estimate the success of blinding (Table 2).

**Table 2:** **Data collection**

**Outcomes**	**Measures**	**Follow-up**
Adherence to antihypertensive or statin therapy	Prescription of antihypertensive drugs and statins (patient record)	T3
	Intake of antihypertensive medication or statins (structured telephone interview)	T3
Informed choice	Knowledge	T1
	Treatment goals: Statin choice, levels of blood pressure and HbA1c, and smoking	T1
	Achievement of goals regarding statins, blood pressure, HbA1c, and smoking	T3
Achievement of goals	Treatment goals: Statin choice, levels of blood pressure and HbA1c, and smoking as well as patient’s prioritised treatment goal (patient record)	T2
	Statin intake (structured telephone interview)	T3
	Blood pressure (standardised measure)	T3
	HbA1c level (laboratory value)	T3
	Smoking cessation (structured telephone interview)	T3
Prioritisation	Prioritised treatment goal: Statin choice, blood pressure, HbA1c or smoking cessation	T1, T2
Realistic expectations	Questionnaire developed based on the contents of the DA and the teaching module	T1
Knowledge	Questionnaire developed on the basis of Bloom’s taxonomy [65] and evidence-based information on heart attack prevention in type 2 diabetes [33]	T1

T1 = at the end of the teaching session; T2 = during physician consultation; T3 = at 6 months follow-up.

### Data synthesis

All statistical analyses are carried out according to the intention-to-treat principle. Missing data will be imputed using the method of multiple imputation if feasible. All analyses will be computed using IBM SPSS Statistics 22.0 for Windows.

Baseline characteristics are described using means of standard deviation (± SD) or frequencies, as appropriate according to the level of measurement.

#### Primary outcome

Fisher’s exact test will be used to compare the groups regarding the adherence to antihypertensive or statin therapy.

#### Secondary outcomes

Fisher’s exact test will be used to compare the groups regarding 1) the informed choice regarding statin treatment, smoking, blood pressure and HbA1c; 2) rates of individual goal achievement (yes/no): statin choice, smoking status; and 3) rates of changed treatment goals.

Unpaired t-tests will be used to compare the study groups regarding 1) the average difference between planned and achieved values of blood pressure; 2) the average difference between planned and achieved level of HbA1c; and 3) the level of realistic expectations and knowledge.

Mann–Whitney *U*-test will be used to compare the medication prescription from T0 to T2, T3 (increase/unchanged/decrease).

Unpaired t-tests will be used to compare the study groups regarding 1) the change of clinical parameters (HbA1c, blood pressure, cholesterol) from T0 to T3 and 2) the mean difference between perceived risk and assessed risk between groups.

Difference in the chosen prioritised treatment goals between groups will be compared by using chi-square test.

### Sample size

We hypothesise patients in the ISDM group to reach higher adherence to prescriptions of antihypertensive and statin medication.

Based on current DMP-data [39] and the preliminary results of the pilot RCT [35], we assume that approx. 60% of patients in the control group will adhere to the choice. We estimate that more than 80% of patients in the intervention group will adhere.

We calculate the sample size providing 80% power to detect an absolute difference of 20% between the intervention and the control group, using Fisher’s exact test at the 5% level of significance. Thus, in a non-cluster RCT data on about 90 patients per group (180 participants) would need to be included in data analysis.

Since observations on individuals in the same cluster tend to be non-independent, the effective sample size is less than the total number of individual participants. The reduction in effective sample size depends on the average cluster size (m) and the degree of correlation within clusters (intracluster correlation coefficient, ICC). To retain power, the sample size should be multiplied by the so-called design effect (DE = 1 + (m - 1) × ICC) [36]. However, we did not identify data, allowing an estimation of a probable ICC for the primary endpoint used in our study. Krones et al. [28] reported an ICC of 0.06 for an endpoint relating to patient participation. Branda et al. [63] published an ICC of 0 or nearly 0 for knowledge, decisional comfort, and medication adherence. Thus estimating an ICC of 0.03 and a mean cluster size of 13 participants, we calculate with a design-effect of DE = 1.36. Hence, data on 122 patients per study arm (total 244 participants) need to be included in data analysis. Estimating a drop-out rate of about 20%, 306 participants will be recruited for randomisation, distributed across 24 clusters (practices). This power calculation was conducted by Thomas Lehmann Biostatistician (Jena, Germany).

### Process evaluation

The effectiveness of the ISDM programme may depend on the complex interaction between components (e.g. provider training module and educational strategies) and conditions of the setting.

Based on the framework by Grant et al. [64], the underlying processes involving clusters and patients will be monitored to explore barriers and promoting factors of the implementation of the intervention programme. Field exploration has indicated possible barriers regarding extra workload and high organisational efforts. Telephone interviews with MAs of the intervention group will be conducted after all patients attended the teaching sessions. Interviews will focus on possible barriers in the implementation of the teaching module, organisational aspects, and team collaboration. At the end of the study, interviews with the GPs will be conducted to assess the perceived effort for the practices.

The standardised telephone interviews will be used to assess the primary endpoint. The interviews will also focus on barriers of adherence and treatment effort perceived by the patients. To avoid that they feel kept under surveillance and to avoid socially desirable answers, questions regarding statin adherence will be embedded into more general questions e.g. on individual treatment effort. All interviews will be recorded and qualitatively analysed. Results will be used to plan the optimisation of implementation concept.

### Ethical approval

The study protocol was approved by the ethics committee of the University Hospital Jena and the Medical Council of the Free State of Thuringia. Written participant information about study objectives and procedures are given to eligible patients. Standardised forms are used to document informed consent.

### Confidentiality

In order to maintain data privacy, pseudonyms are used to combine data sets (baseline and follow-up data) and to identify data if patients withdraw informed consent. The pseudonym list of practices in the Free State of Thuringia is kept under lock at the University Hospital Jena, the list of practices in Hamburg at the Unit of Health Sciences and Education of the University of Hamburg.

## Discussion

In this cRCT, a novel SDM intervention will be compared with usual care in the setting of primary diabetes care.

The study has several strengths. The intervention is intended to promote the implementation of current guideline recommendations into practice, by systematically guiding patients together with their healthcare providers to deliberate on options and to set individual treatment goals in terms of SDM [1,8,20]. Patient teaching module and provider training are brief in their duration, which simplifies implementation into practice. Preliminary studies [32,33,35] have indicated good acceptance and feasibility.

The study has limitations. Firstly, it is impossible to keep the practices blind. At a kick-off symposium MAs and physicians have received information on the innovative ISDM approach to motivate them for participation in the study. Secondly, cluster designs in particular are susceptible for selection bias. This problem will be addressed by recruiting patients before randomisation. Thirdly, since it is not feasible to reliably assess SDM in the context of this study, we decided to use adherence to prescribed medication as an indirect measure. Similar to SDM, adherence is a behaviour influenced by subjective norms such as perceived attitudes of other individuals. Self-report can be biased by socially desired answers and pill count as a more objective measure is not feasible within this study. In order to avoid interfering with the intervention we will not assess self-reported adherence at baseline. Rather, medication prescriptions will be used as a surrogate to evaluate comparability of groups at baseline.

Fourthly, in order to reduce complexity we focused on the most relevant cardiovascular complication in type 2 diabetes, i.e. myocardial infarction. However, prevention requires a holistic approach. Issues such as stroke or microvascular complications are already addressed in the standard treatment and teaching programmes within the DMP, without however including numerical and comparative risk information. In order to communicate benefits and harms of blood glucose and blood pressure control we used UKPDS data [5,52] on the combined endpoint *any diabetes-related endpoint* which also includes other cardiovascular outcomes.

At the end of this study, information on the effectiveness of the ISDM programme and on processes that interfere with or promote a successful implementation into primary care practices will be available.

## Abbreviations

ADA: American diabetes association
cRCT: Cluster randomised controlled trail
CVD: Cardiovascular disease
DA: Decision aid
DE: Design effect
DMP: Disease management programme
EASD: European association for the study of diabetes
EBPI: Evidence-based patient information
EFSD: European foundation for the study of diabetes
GP: General practitioner
ICC: Intracluster correlation coefficient
ICD: International classification of diseases
ISDM: Informed shared decision making
MA: Medical assistant
RCT: Randomised controlled trial
SD: Standard deviation
SDM: Shared decision making
ZI: Central research Institute of ambulatory health care
